# Facile Synthesis of g-C_3_N_4_/TiO_2_/Hectorite Z-Scheme Composite and Its Visible Photocatalytic Degradation of Rhodamine B

**DOI:** 10.3390/ma13225304

**Published:** 2020-11-23

**Authors:** Rong You, Jinyang Chen, Menghan Hong, Jinrui Li, Xiaomin Hong

**Affiliations:** School of Environmental and Chemical Engineering, Shanghai University, Shanghai 201900, China; Mohan@shu.edu.cn (M.H.); 975904228@shu.edu.cn (J.L.); HXM1102@shu.edu.cn (X.H.)

**Keywords:** Z-scheme, oxygen vacancy, photo-catalysis, hectorite, g-C_3_N_4_

## Abstract

A novel g-C_3_N_4_/TiO_2_/hectorite Z-scheme composites with oxygen vacancy (Vo) defects and Ti^3+^ were synthesized by so-gel method and high temperature solid phase reaction. This composite exhibited high visible photo-catalytic degradation of rhodamine B (RhB). The apparent rate constant of g-C_3_N_4_/TiO_2_/hectorite was 0.01705 min^−1^, which is approximately 5.38 and 4.88 times that of P25 and g-C_3_N_4_, respectively. The enhancement of photo-catalytic efficiency of the composites can be attributed to the great light harvesting ability, high specific surface area and effective separation of electrons(e^−^) and holes(h^+^). The F element from Hectorite causes the formation of Vo and Ti^3+^ in TiO_2_, making it responsive to visible light. The effective separation of e^−^ and h^+^ mainly results from Z-scheme transfer of photo-produced electrons in g-C_3_N_4_/TiO_2_ interface. The composites can be easily recycled and the degradation rate of the RhB still reached 84% after five cycles, indicating its good reusability.

## 1. Introduction

The dyestuff industry discharges organic sewage containing dyes and intermediate products. The organic pollutants will damage the ecological environment and cause teratogenic and carcinogenic effects in humans [1]. Owing to light sensitivity, the photo-catalytic degradation of the wastewater has been gaining attention. Fujishima and Honda [2] (1972) first discovered that TiO_2_ can decompose water under ultraviolet light and thus a growing number of studies have focused on photo-catalysis in the treatment of wastewater [3,4]. 

Titanium dioxide exhibits low toxicity, low cost, high chemical and physical stability and photo-degradation of some organic pollutants in water [5]. However, TiO_2_ has some limitations such as low surface area and porosity, low utilization rate of sunlight (only 4% can be used), high aggregation and poor reusability [6]. Therefore, it is of great significance to prepare recyclable photo-catalysts with good adsorption and higher catalytic performance.

Clay has a large specific surface area and special layered structure. Some clay materials can change the phases of the semiconductor or improve the separation of e^−^ and h^+^ [7]. Therefore, it should be a good support. Hectorite (or laponite) is a kind of layered clay material, which has good dispersibility, thixotropy, adsorption and ion exchange ability. The molecular formula of synthetic hectorite is M_x_[Li_x_Mg_6-x_Si_8_O_20_(OH)_y_F_z_] (M=Na, Li). It is known that hectorite can form a house of cards structure in water with exfoliated discrete plates of 20–30 nm, which is suitable for the synthesis of catalyst composites [8,9,10,11]. Titanium incorporated into hectorite has small crystal sizes, large porosity and high specific surface area [12,13,14]. However, due to the wide band gap of TiO_2_, TiO_2_/hectorite composites are considered to only have efficient photo-catalytic activity in ultraviolet light. It has been reported that F ion doped in TiO_2_ can cause the generation of Vo and Ti^3+^, which makes TiO_2_ respond to visible light [15,16,17,18]. Therefore, it is possible that the F element from synthetic hectorite introduces Vo and Ti^3+^ into TiO_2_ by combining TiO_2_ with hectorite and making it exhibit photocatalytic ability under visible light.

Non-metallic g-C_3_N_4_ has been found to have excellent visible light absorption in recent years [19,20,21]. In addition, it has the advantages of good chemical and thermal stability, low cost, water resistance and biocompatibility [22]. However, its photo-catalytic efficiency is limited by limited separation efficiency of photogenic electrons and holes, narrow spectral response, low surface area and so on [23,24,25]. Recently, the hetero-junction catalyst g-C_3_N_4_/TiO_2_ has been studied for the promoted separation efficiency of photogenic e^−^ and h^+^ due to the well matched energy structure [26,27,28].

Therefore, it is feasible to synthesize a ternary photo-catalyst by combining g-C_3_N_4_ with TiO_2_/hectorite to construct a photo-catalytic degradation of visible light. In this article, a g-C_3_N_4_/TiO_2_/hectorite Z-scheme composite with Vo and Ti^3+^ was synthesized. The photo-catalytic degradation of RhB was carried out in visible light irradiation and a possible photocatalytic mechanism was proposed.

## 2. Experimental

### 2.1. Materials

The synthetic hectorite (Na_0.66_[Mg­_5.34_Li_0.66_Si_8_O_20_(OH·F)_4_]) was obtained from Jufeng New Material Technology Co. (Anhui, China). Sinopharm Chemical Reagent Co. (Shanghai, China) provided tetra-butyl titanate (C_16_H_36_O_4_Ti, TBOT), hydrochloric acid (HCl), urea (CH_4_N_2_O) and rhodamine B (C_2_­_8_H_31_ClN_2_O_3_, RhB). Isopropanol (IPA), 1,4-benzoquinone (BQ), edetate disodium (EDTA-2Na), sodium nitrite (NaNO_2_) and ethanol (C_2_H_5_OH) were purchased from Alighting Reagent Co. (Shanghai, China). All of them were analytical reagent grade. Degussa P25 was purchased form Evonik Industries AG (Frankfurt, Germany). The water used in the experiment was distilled water. 

### 2.2. Catalyst Preparation 

#### 2.2.1. g-C_3_N_4_

Carbon nitride powders were synthesized according to the previous report [29]. About 10 g of urea was put into a covered crucible in a furnace at room temperature. Then it was heated to 550 °C at 10 °C/min, heated for 2 h, cooled to room temperature and ground into powder.

#### 2.2.2. TiO_2_/Hectorite Composites

Firstly, about 1.3 g hectorite was added into 65 mL distilled water with stirring for 2 h to form a suspension. Secondly, 8 mL of TBOT was added in 17 mL ethanol with stirring for about 30 min to obtain a light yellow solution. Then a solution of 1.4 mL distilled water, 8 mL anhydrous ethanol and 0.75 mL HCl (65%) was added into the above light yellow solution drop by drop for 1 h to obtain a transparent TiO_2_ sol. The transparent sol was slowly added to the hectorite suspension with stirring for 2 h at 40 °C to form a slurry. The added amount of TiO_2_ was kept to 18 mM of 1 g hectorite and the pH of the slurry was about 3. The slurry was washed and centrifuged for several times, aged for 18 h, dried at 80 °C and finally calcined at 450 °C for 2.5 h.

#### 2.2.3. g-C_3_N_4_/TiO_2_/Hectorite Composites

About 0.05 g g-C_3_N_4_ and 0.1 g synthesized TiO_2_/hectorite composite were ground for 30 min and then the well mixed powders were calcined at 500 °C for 30 min. 

### 2.3. Characterizations

X-ray diffraction (XRD) was performed on a X′Pert3 Powder X-ray diffractometer (PANalytical, Almelo, The Netherlands) by using Cu-Kα radiation (45 kV, 40 mA). The scanning speed was 2°/min, the step length was 0.01314° and the scanning range of 2θ was 10–80°. The scanning electron microscopy (SEM) images were recorded on a S-4800 scanning electron microscope (Hitachi, Tokyo, Japan) equipped with an energy dispersive spectroscope (5.0 kV). Ultraviolet-visible diffuse reflectance spectra (UV-Vis DRS) were detected at ambient by a Lambda 950 UV-Vis spectrophotometer (Perkin-Elmer, MA, USA) equipped with an integrating sphere and BaSO_4_ was used as standard. The N_2_ adsorption-desorption isotherms were determined on an ASAP 2020M nitrogen adsorption apparatus (Micromeritics, Norcross, GA, USA) at liquid nitrogen temperature (77 K). Photoluminescence (PL) spectra were measured on the FLS980 spectrometer (Edinburgh Instruments, UK) with an excitation wavelength of 360 nm. X-ray photoelectron spectroscopy (XPS) was determined on an Escalab 250 Xi electron spectrometer (Thermo Fisher Scientific, Hillsboro, OR, USA) by using 15 kV Al-Kα X-ray radiation at 150 W. The binding energies were calibrated according to the carbonaceous C1s at 284.6 eV.

### 2.4. Photo-Catalysis

The visible photo-catalysis of g-C_3_N_4_/TiO_2_/hectorite was investigated by degrading RhB in a self-made reactor. The photo-catalytic degradation was performed under a 500 W Xenon lamp with 1 M NaNO_2_ as 400 nm cut-off filter solution [30]. Aeration was supplied to facilitate the mixture of the catalyst and RhB solution. The lamp was kept approximately 4 cm away from the solution.

About 1 mg of the catalysts was dispersed in 30 mL of 10 ppm RhB solution. Before the irradiation, a dark reaction was conducted for 30 min on the surface of the composites to achieve an adsorption-desorption equilibrium. About 2 mL of suspension was sampled in the same interval and the photo-catalyst was removed from the solution by filtration with a 0.45 μm filter membranes. To evaluate the photocatalytic efficiency, WF Z UV-2800H UV–vis spectrophotometer (Unico, Suite E Dayton, NJ, USA) was used to measure the absorbance of the solution at 553 nm (RhB).

## 3. Result and Discussion

### 3.1. Characterization

#### 3.1.1. X-ray Diffraction (XRD)

**Figure 1:**
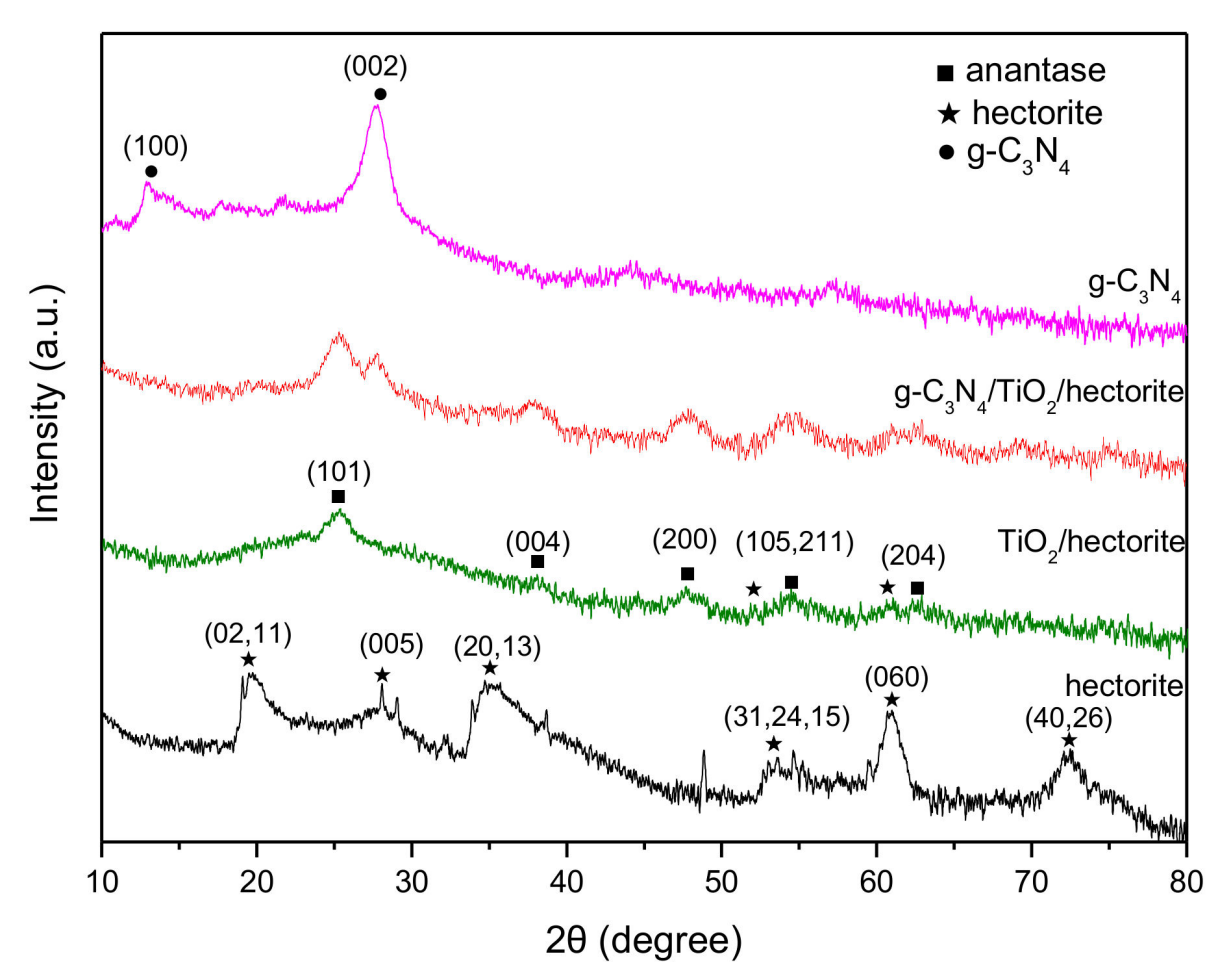
X-ray diffraction (XRD) patterns for hectorite, TiO_2_/hectorite, g-C_3_N_4_/TiO_2_/hectorite and g-C_3_N_4_.

The XRD patterns of hectorite, TiO_2_/hectorite, g-C_3_N_4_ and g-C_3_N_4_/TiO_2_/hectorite are shown in Figure 1. Carbon nitride shows the highly crystalline and has two characteristic peaks at 12.9 and 27.6°, corresponding to structural packing arrangement and inter planar stacking of aromatic ring, respectively (Joint Committee on Powder Diffraction Standards (JCPDS) Card No. 87-1526) [31].

Hectorite has diffraction peaks of 2θ approximately at 19.6, 28.0, 35.1, 53.3, 61.0 and 72.3° according to references [32,33]. The widened reflections indicate low crystallinity and small particle size. However, as for the synthesized TiO_2_/hectorite and g-C_3_N_4_/TiO_2_/hectorite, the characteristic peaks of hectorite become weak and a new peak appears at 20–30° resulting from the present of amorphous silicon [34]. It has been reported that for hectorite, magnesium from magnesium oxide octahedron is more easily replaced by ions, while silicon from silica tetrahedron is difficult [35]. The atomic ratio of Mg/Si is about 0.56 for g-C_3_N_4_/TiO_2_/hectorite measured by XPS (Table 1), while 0.67 for hectorite calculated by a molecular formula, indicating that only about 16.42% of magnesium ions are leached out. Therefore, the main framework of hectorite still remains.

Anatase TiO_2_ shows characteristic peaks at 25.3, 37.8, 48.1, 54.0, 55.1 and 62.8°, which correspond to the crystal planes of (101), (004), (200), (105), (211) and (204), respectively (JCPDS Card No. 21-1272). The titania containing samples TiO_2_/hectorite and g-C_3_N_4_/TiO_2_/hectorite show the presence of crystalline anatase phase. The crystallite sizes of TiO_2_ in TiO_2_/hectorite and g-C_3_N_4_/TiO_2_/hectorite are 11.0 and 11.3 nm, respectively, according to the most obvious (101) peak by the Debye-Scherrer equation [10] (Table 2). It indicates that addition of g-C_3_N_4_ takes no obvious effect on the crystal size of TiO_2_. However, the stronger peak intensity of g-C_3_N_4_/TiO_2_/hectorite indicates the higher crystallinity compared with TiO_2_/hectorite. 

#### 3.1.2. Scanning Electron Microscopy (SEM)

**Figure 2:**
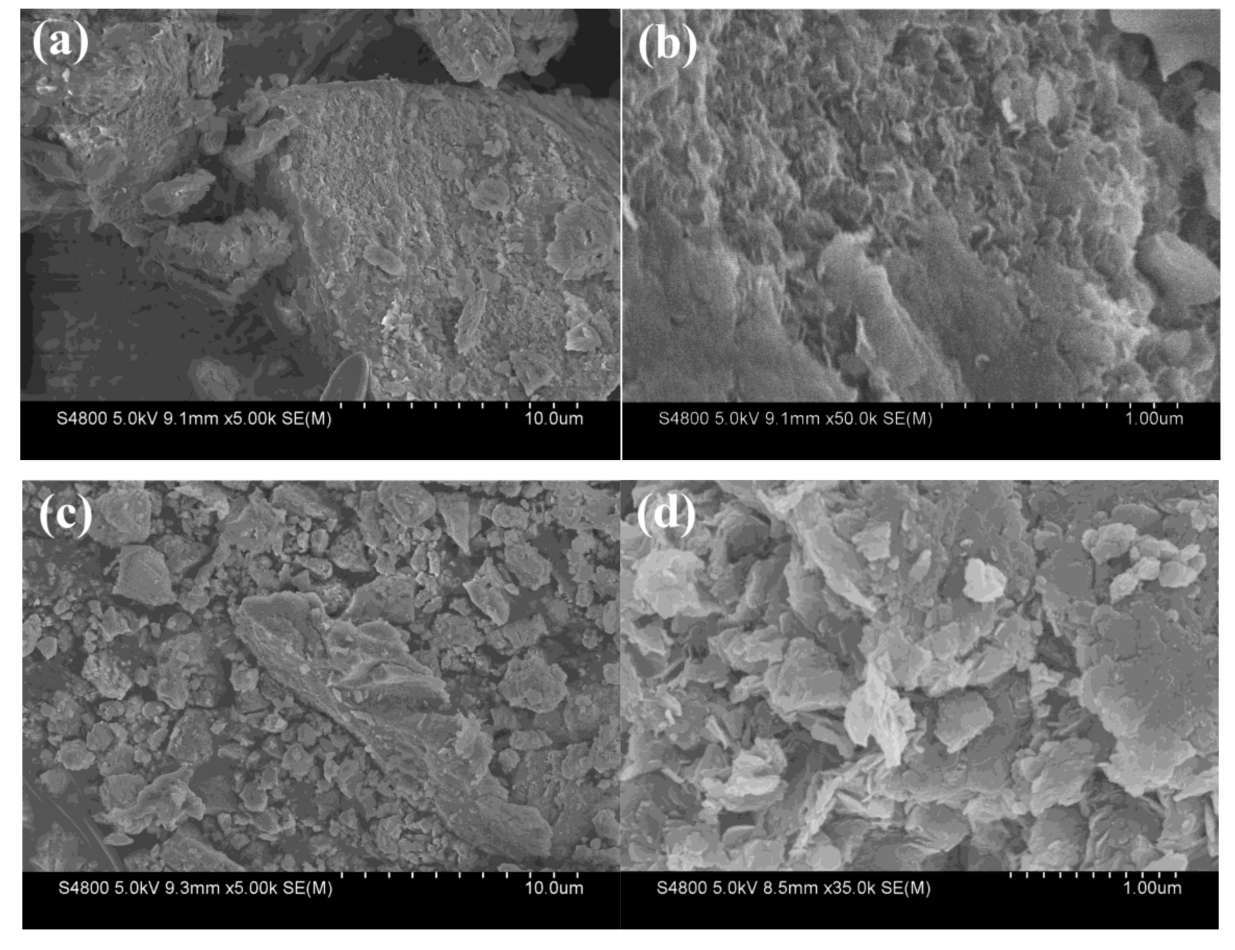

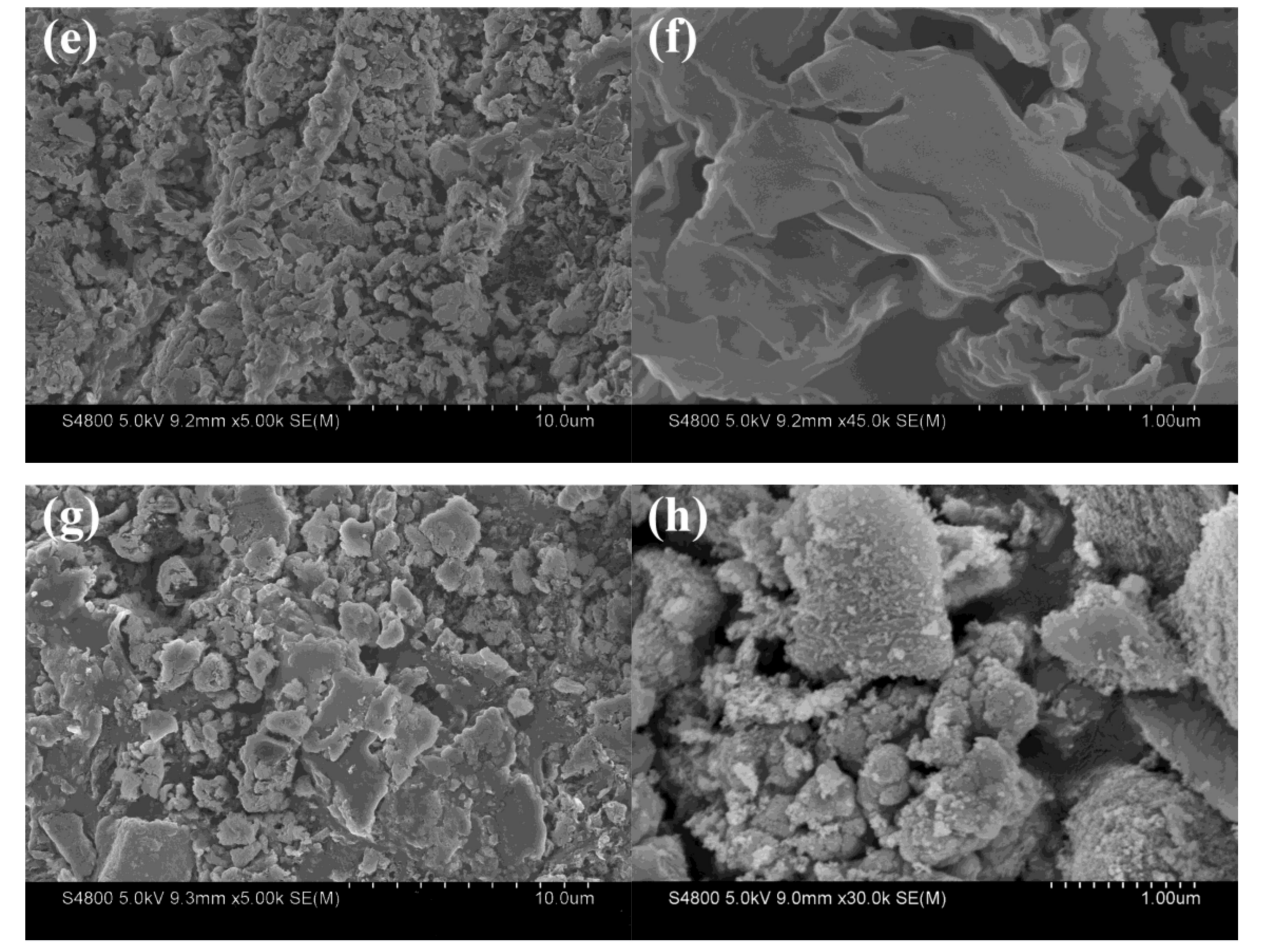
Scanning electron microscopy (SEM) images:(**a**,**b**) are hectorite; (**c**,**d**) are TiO_2_/hectorite; (**e**,**f**) are g-C_3_N_4_; (**g**,**h**) are g-C_3_N_4_/TiO_2_/hectorite.

SEM images of hectorite, TiO_2_/ hectorite, g-C_3_N_4_ and g-C_3_N_4_/TiO_2_/hectorite are shown in Figure 2. Figure 2a,b show that hectorite are blocky in shape with compact layered structure. However, after being pillared by TiO_2_, the particle size of hectorite is significantly decreased, showing a looser structure with disordered accumulation of the lamellas as shown in Figure 2c,d, which benefits to increase the specific surface area and improve the photo-catalytic activity. The layered structure indicates that the framework of hectorite is less damaged, which is consistent with the XRD analysis. The result indicates that TiO_2_ particles successfully intercalate into the layers of hectorite. Figure 2e,f show a porous structural material composed of plenty of particles of g-C_3_N_4_. The particles are formed by accumulation of smooth lamellas. Figure 2g,h give that g-C_3_N_4_/TiO_2_/hectorite are particles of different sizes with a rough surface. Compared with TiO_2_/hectorite and g-C_3_N_4_, the morphology of g-C_3_N_4_/TiO_2_/hectorite changes significantly, which indicates that a ternary heterogeneous system is successfully constructed.

#### 3.1.3. N_2_ Adsorption-Desorption Isotherms

**Figure 3:**
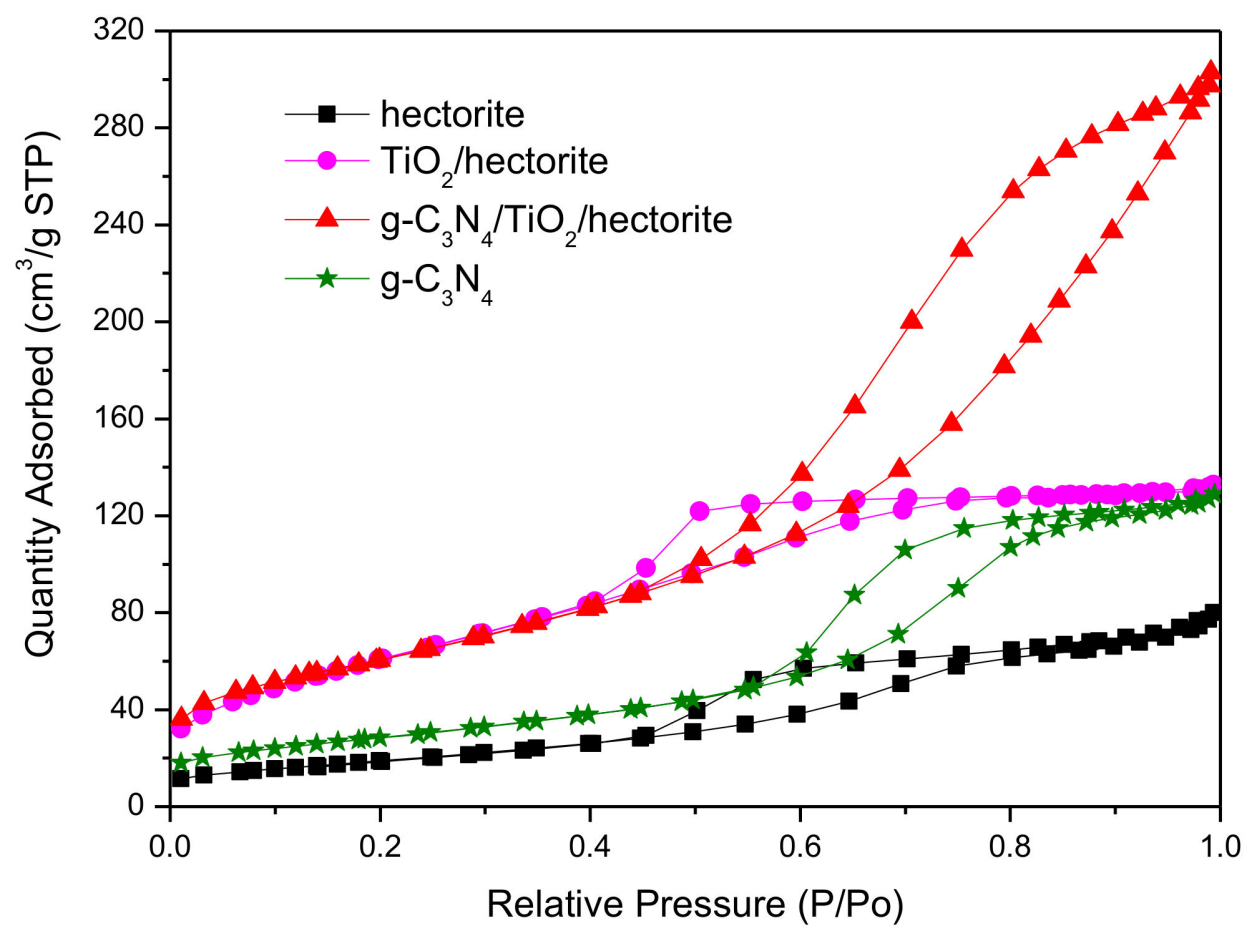
N_2_ adsorption and desorption isotherms of hectorite, TiO_2_/hectorite, g-C_3_N_4_ and g-C_3_N_4_/TiO_2_/hectorite.

Figure 3 shows the N_2_ adsorption-desorption isotherms of hectorite, TiO_2_/ hectorite, g-C_3_N_4_ and g-C_3_N_4_/TiO_2_/hectorite. All the isotherms are type IV adsorption isotherms, which correspond to mesoporous structure. The N_2_ adsorption capacities are obviously higher than 0 cm^3^/g at the relative pressure of 0, indicating abundant micropores in the samples. The adsorption isotherms of hectorite and TiO_2_/hectorite exhibit H2 hysteresis loops, which correspond to interlayers with bottle necks and contractions or a complex network of interconnected pores [36]. The hysteresis loop of g-C_3_N_4_ samples is close to the H1 hysteresis loop, which is relative to the porous structure consisted by particles or spheres [34]. It is consistent with the SEM image of g-C_3_N_4_. g-C_3_N_4_/TiO_2_/hectorite nano-composite shows characteristics of the H3 loop, corresponding to the mesoporous and microporous structures. 

The pore volume, pore diameter, specific surface area and crystallite size of samples are summarized in Table 2. Compared with TiO_2_/hectorite, the pore size and volume of g-C_3_N_4_/TiO_2_/hectorite are increased greatly, which would be conducive to the adsorption and degradation of organic contamination. This might be ascribed to the intercalation of some g-C_3_N_4_ into the hectorite layers or the formation of a more mesoporous structure and the further exfoliation of g-C_3_N_4_ due to the thermal etching effect [37]. Besides, the specific surface area of g-C_3_N_4_/TiO_2_/hectorite composites reaches 219.0311 m^2^/g, which is about twice that of pure g-C_3_N_4_. This indicates that TiO_2_/hectorite has an effective role on increasing the specific surface area of the composites, which is conducive to providing more active sites and is of more favorable for photo-catalytic reaction.

#### 3.1.4. Ultraviolet–Visible Diffuse Reflectance Spectra(UV-Vis DRS)

**Figure 4:**
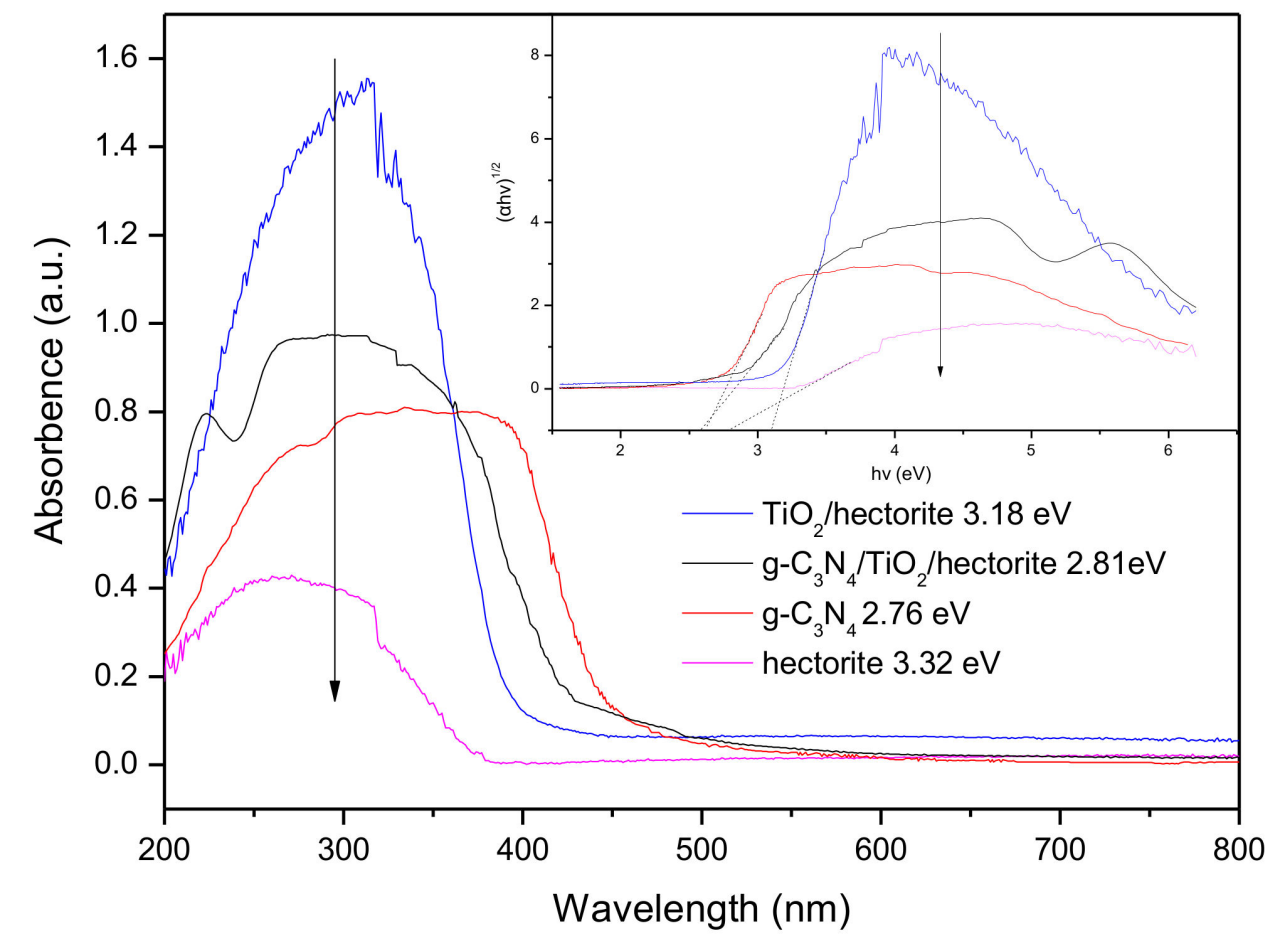
Ultraviolet–visible (UV–Vis) diffuses reflectance absorbance spectra and band gaps of hectorite, TiO_2_/hectorite, g-C_3_N_4_/TiO_2_/hectorite and g-C_3_N_4_.

Figure 4 is the UV-Vis DRS and the band gaps of g-C_3_N_4_, TiO_2_/hectorite and g-C_3_N_4_/TiO_2_/hectorite. The band gaps were calculated by the equation (Equation (1)):

(1) $$\alpha h\nu =A{\left(h\nu \;-\;E_{g}\right)}^{1/2}$$

where *α*, *h*, *ν*, *A* and *E_g_* are the optical absorption coefficient, Planck constant, photon frequency, a constant and band gap, respectively [38]. Figure 4 shows that the light absorption ability of hectorite is weak from 200 to 800 nm wavelength range, which might be due its component and small particle size. Its band gap is 3.33 eV. After incorporated TiO_2_, TiO_2_/hectorite absorbs ultraviolet strongly. The band gap of TiO_2_/hectorite is 3.18 eV, which is close to that of pure TiO_2_ (3.2 eV). The absorption edge of g-C_3_N_4_ is about 449 nm and the band gap is 2.76 eV.

It can be observed that the absorption edge of g-C_3_N_4_/TiO_2_/hectorite extends to 438 nm and shows a strong intensity from 365 to 497 nm compared with TiO_2_/hectorite. Its band gap was about 2.81 eV due to the heterojunction formation of the composite. In addition, the absorbance intensity of the heterogeneous composites presents an enhancement from about 482 to 800 nm compared with g-C_3_N_4_. Therefore, g-C_3_N_4_/TiO_2_/hectorite can enhance the absorption under UV-Vis light irradiation over 400 nm, which give advantages to photo-catalysis under visible light.

#### 3.1.5. Photoluminescence (PL)

**Figure 5:**
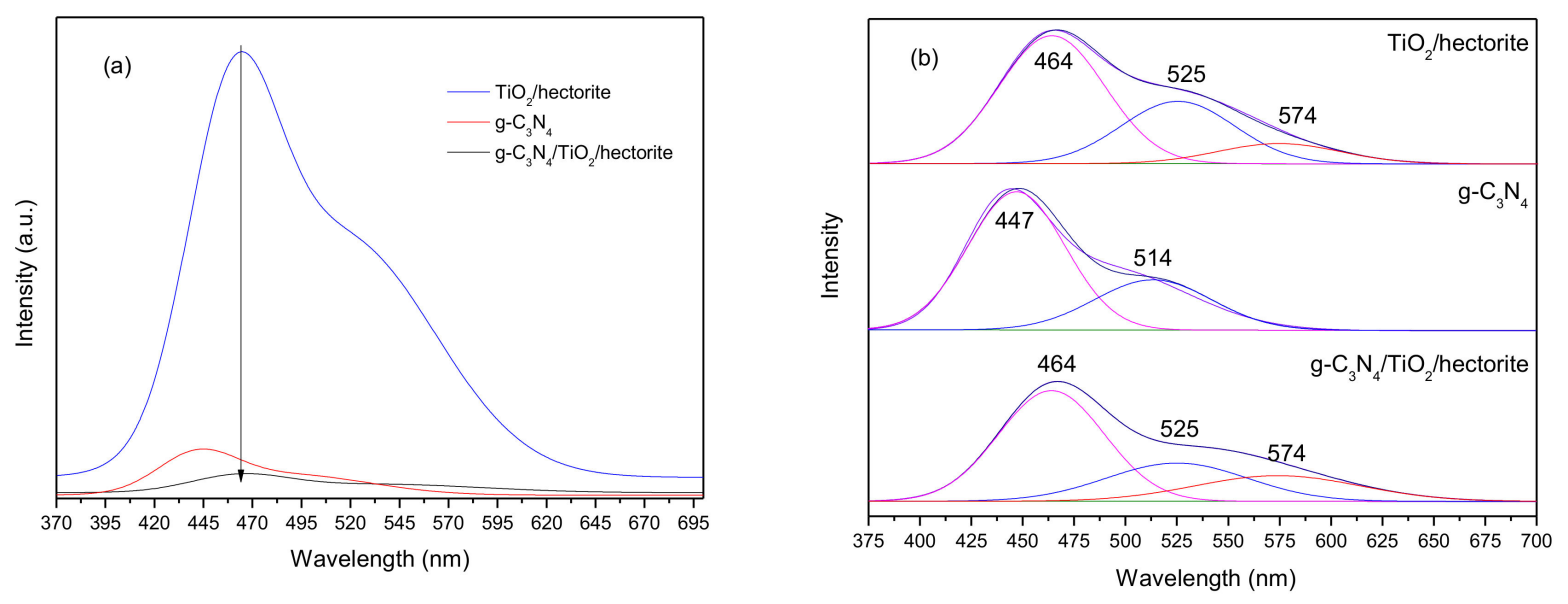
(**a**) Photoluminescence (PL) spectra and (**b**) Gaussian function fitted graphs (R^2^ = 0.996) for TiO_2_/hectorite, g-C_3_N_4_ and g-C_3_N_4_//TiO_2_/hectorite.

Photoluminescence (PL) spectra were obtained to study the separation and recombination of photogenic e^−^ and h^+^. Figure 5 shows the PL spectra and the Gaussian function fitted graphs (R^2^ = 0.996) for g-C_3_N_4_, TiO_2_/hectorite and g-C_3_N_4_/TiO_2_/hectorite [39]. For g-C_3_N_4_, the emission peak at 447nm corresponds to the absorption band in UV-Vis DRS spectrum. The peak at 514 nm may be caused by defects in g-C_3_N_4_.

TiO_2_/hectorite shows a strong emission from about 430–570 nm (Figure 5a). This is possibly related to the defects in TiO_2_ or silica tetrahedra, which are most probably attributed to the formation of Vo in TiO_2_ caused by F doping, amorphous silicon caused by the damaged hectorite or Ti-O-Si bond between TiO_2_ and hectorite [40,41,42]. However, as mentioned above, the framework of hectorite is less damaged and the silica tetrahedron is difficult to be destroyed by ions, which indicate that the silica tetrahedron may contain fewer defects. In addition, it has been reported that the larger Ti^4+^ tetrahedron is almost undistorted in the TiO_2_–SiO_2_ system, suggesting fewer defects caused by the interaction between TiO_2_ and SiO_2_ [42]. Therefore, it could be considered that Vo in TiO_2_ is the main factor.

After fitting by Gaussian function, there are two peaks at around 464 and 525 nm, most probable corresponding to Vo with two trapped electrons (F center) and one trapped electron (F^+^ center) [16,39]. Moreover, a peak appears at 574 nm, and may be assigned to the self-trapped excitons [43]. The peak corresponding to the absorption edge is not observed, which may be related to the formation of local states below the conduction band edge [43]. It is usually considered that the F center exists on the surface of TiO_2_, whereas the F^+^ center is present in bulk phase. Both the surface Vo and the bulk Vo can improve the absorption ability of visible light [44]. However, during the photo-induced charge transfer process, surface defects are advantageous to separate e^−^ and h^+^, while defects in bulk phase take an opposite effect [18]. The content ratio of the F/F^+^/ center is 1.91 by calculating the area of the two peaks, indicating that F center is more than F^+^ center, which will be beneficial to photocatalysis.

g-C_3_N_4_/TiO_2_/hectorite has a significantly lower PL intensity than TiO_2_/hectorite. Moreover, its PL emission intensity decreases significantly from 406 to 538 nm compared to g-C_3_N_4_. Hence, the decrease of PL intensity for g-C_3_N_4_/TiO_2_/hectorite indicates the promoted separation of e^−^ and h^+^ due to the formation of heterogeneous junction. After fitting by Gaussian function, the peaks at 464, 525, 574 nm belonging to TiO_2_ are observed while the peaks at 447 and 514nm corresponding to g-C_3_N_4_ are not found. This may be due to the strong PL emission of TiO_2_, resulting in the inability to observe the weak emission of g-C_3_N_4_. The ratio of F/F^+^ centers is about 2.17, which is close to that in TiO_2_/hectorite, indicating that a short-term calcination process has no obvious influence on the distribution of Vo in g-C_3_N_4_/TiO_2_/hectorite. 

#### 3.1.6. X-ray Photoelectron Spectroscopy (XPS)

**Figure 6:**
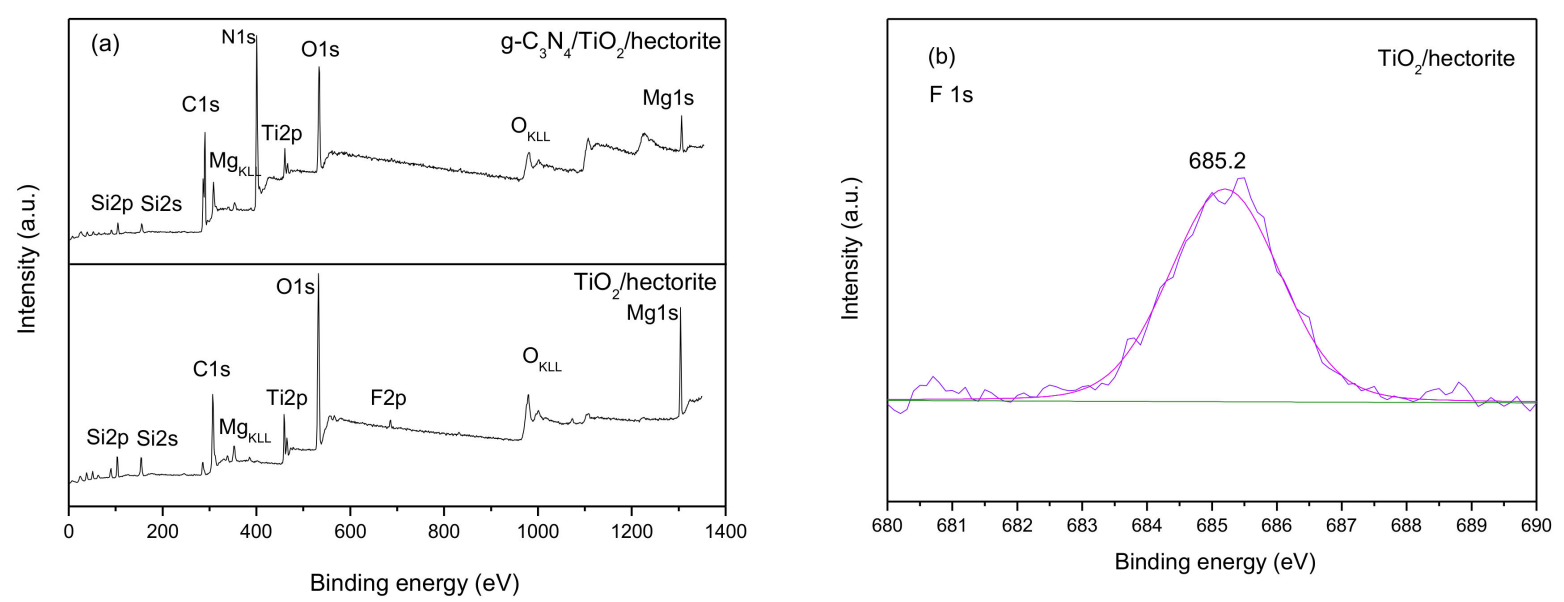

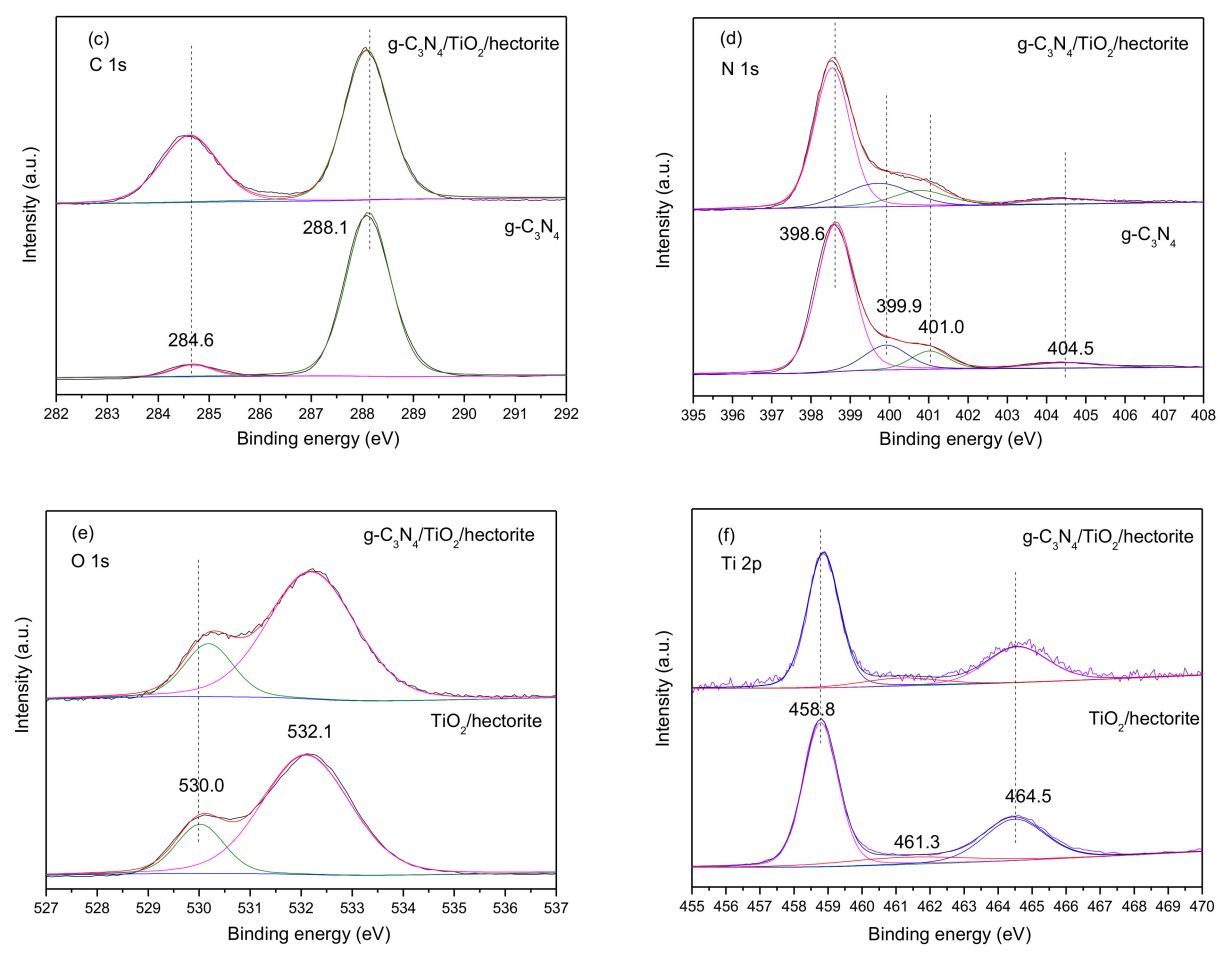
X-ray photoelectron spectroscopy (XPS) spectra of TiO_2_/hectorite, g-C_3_N_4_ and g-C_3_N_4_/TiO_2_/hectorite composite sample: (**a**) is survey spectrum; (**b**) is F 1s; (**c**) is C 1s; (**d**) is N 1s; (**e**) is O 1s; (**f**) is Ti 2p.

Figure 6 is the XPS spectra and they give the elemental states and surface components. C, F, O, Si, Mg and Ti appear in TiO_2_/hectorite while C, N, O, Si, Mg and Ti appear in g-C_3_N_4_/TiO_2_/hectorite (Figure 6a). The disappearance of the element F in g-C_3_N_4_/TiO_2_/hectorite may be due to the low content below the detection limit. Li or Na ions originally existed in hectorite are not observed in TiO_2_/hectorite and g-C_3_N_4_/TiO_2_/hectorite, indicating that ion exchange reactions occur during the preparation process, and these ions may be replaced by hydrated titanium ions or hydrogen ions.

The UV-Vis DRS result of TiO_2_/hectorite shows a intense absorption in the UV region without significant redshift and the PL spectrum shows peaks at 464 and 525 nm corresponding to Vo, which was consistent with previous studies of F-doped TiO_2_ [15,16]. Therefore, the formation of Vo is possibly mainly related to the F-doping. As shown in the spectrum of F 1s for TiO_2_/hectorite (Figure 6b), a peak appears at 685.2 nm, which is related to the physical surface adsorption of F [17].

According to the spectrum of C1s (Figure 6c), the peaks at around 288.1 and 284.6 eV are assigned to sp^2^ hybridized C(-N-C=N) of g-C_3_N_4_ and exogenous sp^2^ hybridized C, respectively [28,29]. As for g-C_3_N_4_/TiO_2_/hectorite, the peaks shift to lower energy region. In the N 1s spectrum of g-C_3_N_4_ (Figure 6d), the peaks at 398.6, 399.9, 401.0 and 404.5 eV can be attributed to sp^2^ hybridized N in -C-N=C or C-N=C=(N_2_C), bridging N in -N-(C)_3_ (N_3_C), N-H_x_ group in the heptazine framework and π-excitations, respectively [37,45]. However, the binding energy of N1s becomes lower in g-C_3_N_4_/TiO_2_/hectorite. 

Seen from the spectrum of O 1s for TiO_2_/hectorite (Figure 6e), the peak at 532.1 eV is mainly associated with the Si-O bond from the silica lattice, while the peak at 530.0 eV may be the Ti-O bond in TiO_2_ [46,47]. Compared with TiO_2_/hectorite, the peak assigned to the Ti-O bond in g-C_3_N_4_/TiO_2_/hectorite shifts to higher position. Surface-adsorbed F (terminal ≡Ti-F group) and surface hydroxyl groups can take a reaction as follows (Equation (2)) [17]:

(2) $$\equiv \mathrm{Ti}-\mathrm{OH}+F^{-}↔\equiv \mathrm{Ti}-F+{\mathrm{OH}}^{-}\;{\;\mathrm{pK}}_{F}=6.2$$

Since acidic conditions are conducive to the adsorption of F and TiO_2_/ hectorite is prepared at a pH of about 3, the surface is occupied more by F. Therefore, no peak corresponding to the surface hydroxyl group was observed.

Two single peaks at 458.8 and 464.5 eV may attributed to Ti 2p_3/2_ and Ti 2p_1/2_ in TiO_2_/hectorite, which resulted from Ti^4+^ in TiO_2_ (Figure 6f) [47]. Peaks of Ti 2p shift to higher binding energy in g-C_3_N_4_/TiO_2_/hectorite. Besides, the peak at 461.3 nm is attributed to the formation of Ti^3+^, which is mainly due to the reduction of T^4+^ by electrons in the adjacent Vo [15,18]. Ti^3+^ is generally considered to be favorable to photocatalysis. Combined with PL analysis, it can be known that the surface adsorption of fluorine causes the generation of Vo and Ti^3+^ (Equation (3)) [15].

(3) $$\left(1-x\right){\mathrm{TiO}}_{2}+{\mathrm{xF}}^{-}\to {\mathrm{Ti}}_{x}^{3+}{\mathrm{Ti}}_{1-x}^{4+}O_{2-x}^{2-}F_{x}^{-}+x/2O^{2--}$$

It is known that higher binding energy means lower electron density [28,47]. The positive shift of O 1s and Ti 2p with the negative shift of C 1s and N 1s suggests that the electrons transfer from TiO_2_ to g-C_3_N_4_. The higher binding energy of Ti 2p_3/2_ (458.8 eV), Ti 2p_1/2_ (464.5 eV) and Ti-O (530.0 eV) of TiO_2_/hectorite compared with pure TiO_2_ (458.4, 464.1 and 529.8 eV) might indicates the electronic migration between TiO_2_ and hectorite owing to the formation of the Ti-O-Si bond [15,47]. This suggests that hectorite benefit the separation of the e^−^-h^+^ pairs. 

### 3.2. Photo-Catalytic Activity

**Figure 7:**
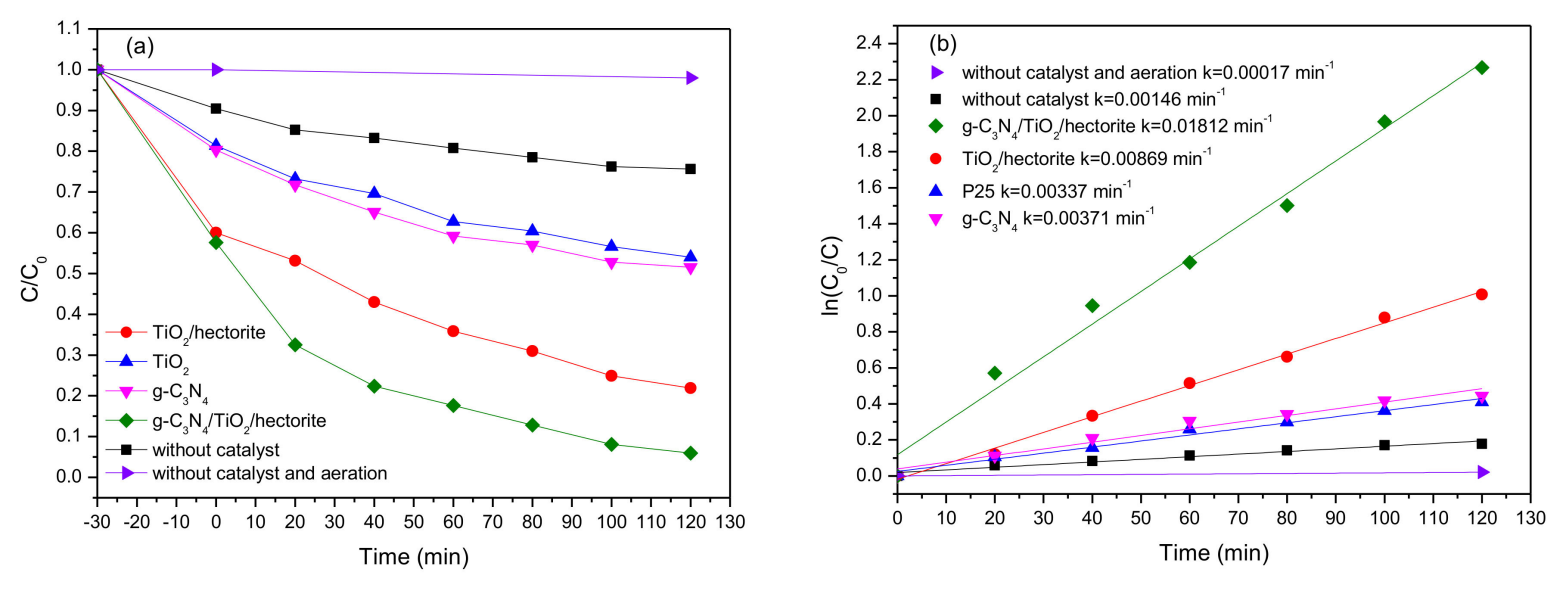
(**a**) Photocatalytic degradation of f rhodamine B (RhB) under visible light; (**b**) kinetic curves of RhB under visible light.

In order to evaluate the activity, RhB was degraded under visible light. Figure 7 gives photo-catalytic degradation and kinetic curves of RhB under visible light. Figure 7a shows that RhB was degraded very little in visible light without a catalyst and aeration. However, with the air involved, the degradation efficiency was up to 24.4% for 2 h, which is mainly caused by oxygen oxidation. After adding g-C_3_N_4_ and P25, 48.0% and 46.0% of removal rates were obtained, respectively. It might be ascribed to the wide band gap of P25 and the low e^−^-h^+^ separation efficiency of g-C_3_N_4_. The removal rate of the TiO_2_/hectorite sample was 78.1%, indicating higher activity compared to P25 and g-C_3_N_4_. As for g-C_3_N_4_/TiO_2_/hectorite, the catalytic degradation rate of RhB reached 94.0% for 2 h irradiation, which is significantly higher than g-C_3_N_4_, TiO_2_ and TiO_2_/hectorite.

The photocatalytic degradation process can be fitted by the equation as follows (Equation (4)):

(4) $$\ln\left(C_{o}/C\right)=\mathrm{kt}$$

where C is the concentration of RhB at certain times (t), C_o_ is the adsorption-desorption equilibrium concentration, and k is the kinetics constant [27]. The photocatalytic activity of the g-C_3_N_4_/TiO_2_/hectorite denoted by the kinetics constant was 0.01705 min^−1^, which is 5.38 and 4.88 times that of P25 and g-C_3_N_4_, respectively (Figure 7b). This indicates that the photocatalytic property of the composite has been improved significantly. The efficient photocatalysis can be ascribed to the high specific surface area, high visible light capture ability and efficient e^−^-h^+^ separation efficiency.

### 3.3. Photocatalytic Mechanism

**Figure 8:**
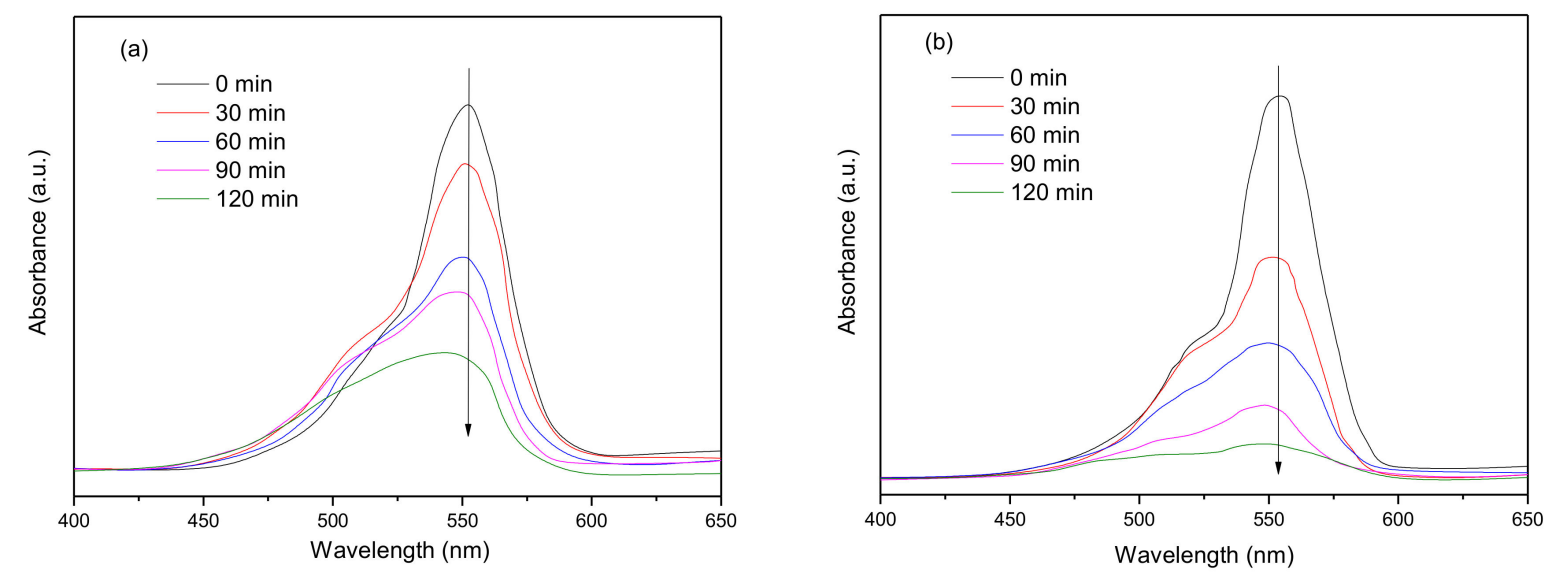
The absorption spectra changes of RhB over time taking place in the presence of (**a**) TiO_2_/hectorite and (**b**) g-C_3_N_4_/TiO_2_/hectorite under visible light irradiation.

To discuss whether the degradation of RhB is mainly due to visible-light catalysis or self-photosensitization [48], the absorption spectra changes of RhB over time in the presence of TiO_2_/hectorite and g-C_3_N_4_/TiO_2_/hectorite are given in Figure 8. When the maximum absorption peak decreases rapidly, the maximum absorption wavelength only undergoes a slight blue shift. This indicates that the ring-opening reaction (deeper oxidation) of benzene ring plays a dominant role in degrading RhB, while the N-dealkylation process takes a secondary place [49]. This means that the degradation of RhB is mainly caused by visible-light catalysis. 

The result indicates that TiO_2_/hectorite is active under visible light, which seems inconsistent with the analysis of UV-Vis DRS. In fact, the absorption spectrum of photocatalyst is a superposition effect of intrinsic and extrinsic absorption bands while UV-Vis absorption spectrum only reflects the intrinsic optical property for the bulk [16]. Therefore, an absorption spectrum could not exactly correspond to the spectral limit of a photocatalytic reaction and the actual band gap should be less than 3.18 eV of TiO_2_ in TiO_2_/hectorite. 

**Figure 9:**
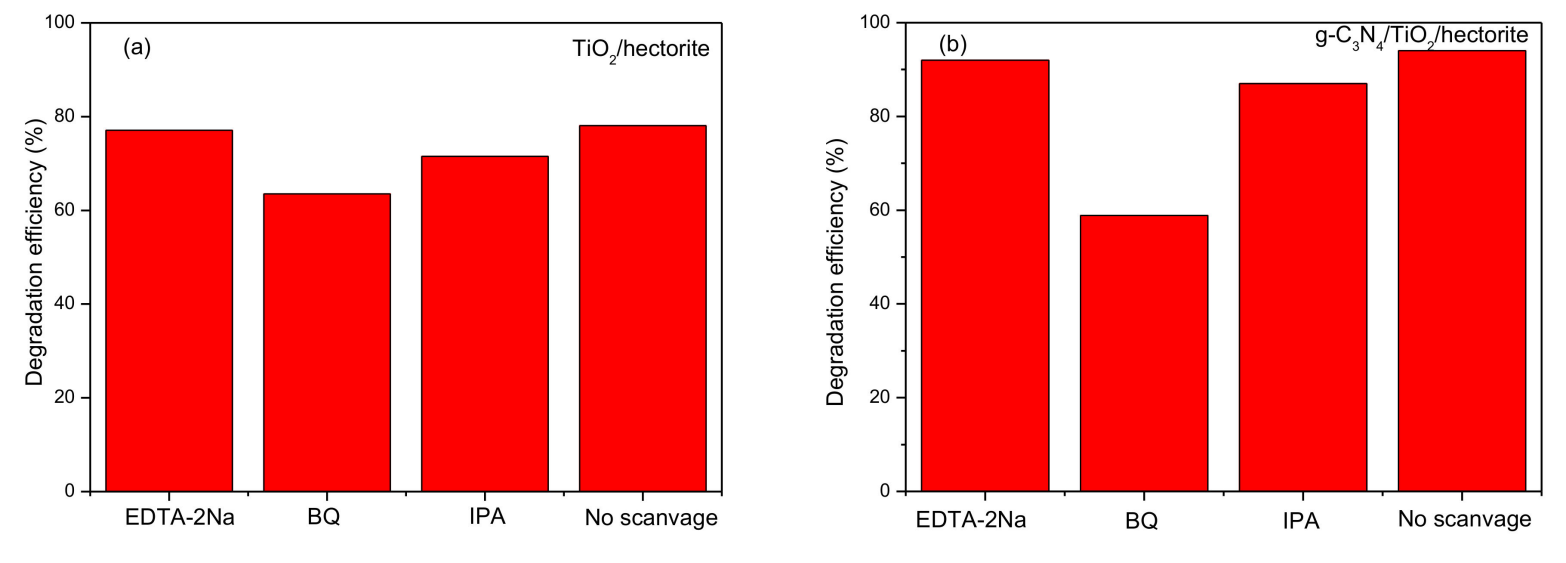
Trapping experiment of active species in the photocatalytic degradation process of RhB over (**a**) TiO_2_/hectorite and (**b**) g-C_3_N_4_/TiO_2_/hectorite composite.

In order to further confirm the electronic migration pathway of composites, trapping experiments were carried out. About 5 mM of BQ, IPA and EDTA-2Na were employed as the scavengers of superoxide radical (·O_2_^−^) and hydroxyl radical (·OH) and h^+^, respectively [27]. Figure 9 gives the trapping results of active species in the photocatalytic degradation process of RhB over TiO_2_/hectorite and g-C_3_N_4_/TiO_2_/hectorite composite, respectively. 

In Figure 9a, the degradation efficiencies over TiO_2_/hectorite have obviously descended after the addition of BQ and IPA, suggesting that the degradation process was mainly related to ·O_2_^−^ and ·OH. The PL analysis proves the existence of Vo, which leads the formation of localized states below the conduction band (CB) of TiO_2_ and the formation of shallow donor states below the CB due to electron redistribution of Ti 3d orbital nearby. The electrons can be excited from the valence band (VB) of TiO_2_ to the Vo and Ti^3+^ under visible light [18]. Vo defects are conducive to the adsorption of O_2_ [18]. Besides, the photocatalytic experiment conducted under the aeration condition provides more O_2_, which further promotes the adsorption of O_2_. The free electrons on Vo defects as well as the photoproduction electrons reduce O_2_ to ·O_2_^−^. XPS shows the presence of surface adsorbed fluorine on TiO_2_. There is a ligand exchange reaction between the surface hydroxyl group and surface adsorbed fluorine and the isoelectric point is about 6.2 (Equation (4)). When pH=9, fluorine adsorption on the surface can be ignored [17]. The photocatalytic experiments were carried out under neutral conditions, so hydroxyl groups and F co-exist on the surface of TiO_2_ (Equation (5)). In bulk solution, the homogeneous free OH radicals (·OH_free_) can be induced by surface adsorbed fluorine (Equation (6)). Surface hydroxyl group reacts with the holes to form ·OH (Equation (7)) [15,17]. In summary, Vo and Ti^3+^ generated from surface adsorbed fluorine may be the main factors for the photocatalytic activity of TiO_2_/hectorite under visible light.

(5) $$\equiv \mathrm{Ti}-\mathrm{OH}+F^{-}\to \;\equiv \mathrm{Ti}-F+{\mathrm{OH}}^{-}$$

(6) $$\equiv \mathrm{Ti}-F+H_{2}O+h^{+}\to \;\equiv \mathrm{Ti}-F+\cdot {\mathrm{OH}}_{\mathrm{free}}+H^{+}$$

(7) $$\equiv \mathrm{Ti}-\mathrm{OH}+H^{+}\to \;\equiv \mathrm{Ti}-{\mathrm{OH}}^{\cdot +}$$

For g-C_3_N_4_/TiO_2_/hectorite, the degradation efficiency of RhB was significantly lower when the BQ was added, while slight descend in the presence of IPA (Figure 9b). It suggests that the degradation process was dominated by ·O_2_^−^ rather than ·OH. Obviously, the catalytic efficiency over g-C_3_N_4_/TiO_2_/hectorite decreased more than that of TiO_2_/hectorite after adding BQ. This can be attributed to the more negative CB potential of g-C_3_N_4_, which is more conducive to reducing O_2_ to ·O_2_^−^. The PL analysis shows that the combination of g-C_3_N_4_ and TiO_2_/hectorite promotes e^−^-h^+^ separation and XPS analysis suggests a transfer from TiO_2_ to g-C_3_N_4_. In addition, XRD shows an obvious 101 surface of TiO_2_ in g-C_3_N_4_/TiO_2_/hectorite, indicating that this surface has more opportunities to interact with g-C_3_N_4_. Previous experimental and theoretical studies had shown that the 101 surface of TiO_2_ and the Vo on TiO_2_ can promote the Z-Scheme process [50,51]. This is mainly due to the fact that the 101 surface of TiO_2_ has a lower energy level and the donor energy level formed by the Vo further increases its Fermi energy level, which promote the separation and transfer of e^-^ from TiO_2_ to g-C_3_N_4_. Combined with the above analysis, it can be concluded that the e^−^-h^+^ transfer may follow a Z-scheme mechanism in g-C_3_N_4_/TiO_2_/hectorite. 

**Scheme 1:**
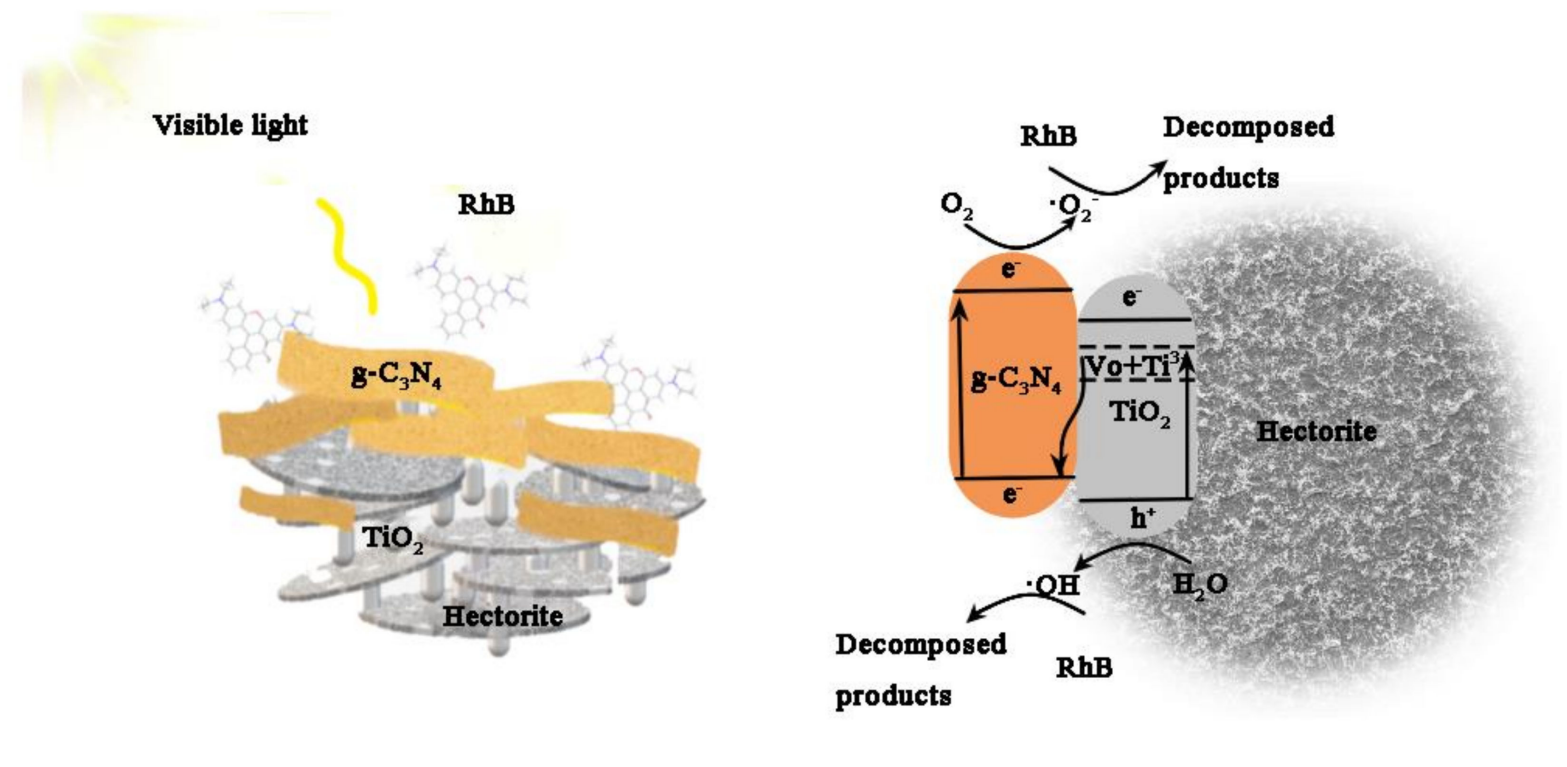
Schematic diagram of the enhancement mechanism for the degradation process under visible light towards g-C_3_N_4_/TiO_2_/hectorite composite.

Based on the results above, a possible photocatalytic mechanism of g-C_3_N_4_/TiO_2_/hectorite is given in Scheme 1. First, the larger surface area of composites provides more active sites, ensuring contact of RhB and catalysis. Then, under irradiation of visible light, the electrons are excited from the VB (2.9 eV vs. Normal Hydrogen Electrode (NHE)) to the Vo and Ti^3+^ in TiO_2_ and from the VB (1.45 eV vs. NHE) to CB (−1.3 eV vs. NHE) in g-C_3_N_4,_ respectively [52,53]. Between the interface of TiO_2_ and g-C_3_N_4_, photo-generated electrons tend to separate and migrate from Vo and Ti^3+^ of TiO_2_ to the VB of g-C_3_N_4_ corresponding to the Z-scheme pathway, which results in the efficient charge separation in the composite photocatalyst. Moreover, hectorite benefits the separation of e^−^-h^+^ pairs. Finally, the electrons stored in CB of g-C_3_N_4_ reduce the absorbed O_2_ to the reactive superoxide radical ion (·O_2_^−^) near the surface of g-C_3_N_4_, which can be ascribed to the fact that CB potential for g-C_3_N_4_ is more negative than the O_2_/·O_2_^-^ potential (−0.28 eV vs. NHE) [16]. The holes in the VB of TiO_2_ react with water molecules (or surface hydroxyls) adsorbed on the surface of TiO_2_ to form ·OH due to the more positive potential of CB than that of ·OH/H_2_O (2.27 eV vs. NHE) [16]. In addition, ·OH_free_ radicals were generated in bulk solution due the fluorine adsorbed on the TiO_2_ surface. Finally, the RhB is oxidized into decomposed products by these main active species ·O_2_^-^ and the subordinate species ·OH. 

### 3.4. Reusability

**Figure 10:**
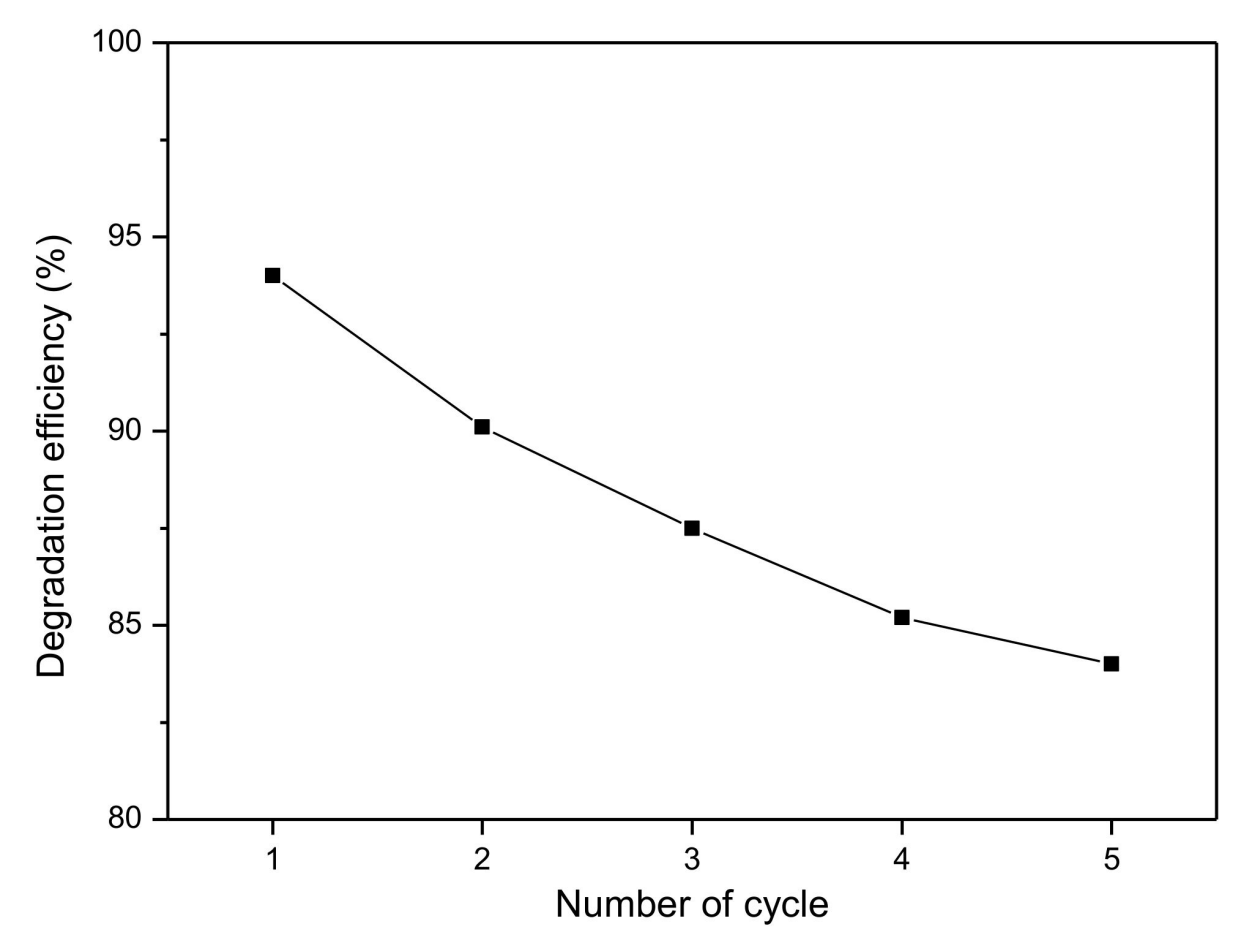
Reusability performance of g-C_3_N_4_/TiO_2_/hectorite composite towards RhB.

The stability and reusability a catalyst is important for its industrial application. The reusability of g-C_3_N_4_/TiO_2_/hectorite composite is given in Figure 10. This shows that after the photo-catalyst has been used 5 times, the decoloring rate remained 84%, indicating that the g-C_3_N_4_/TiO_2_/hectorite has good active stability. The atomic ratio of N/Si decreased from 9.87 to 5.16 after use, which indicates that part of g-C_3_N_4_ is detached from the catalyst (Table 1). This may be mainly related to the agitation in photocatalytic process and centrifugal operation in the recovery process. Moreover, the decrease of the surface adsorption F may also cause the decrease of catalytic activity. Furthermore, g-C_3_N_4_/TiO_2_/hectorite can be easily recovered from the reaction slurry by only settling, whereas as for the P25 and g-C_3_N_4_, the sedimentation is very difficult because of strong suspension. It can be concluded that the composite has good recyclability and stability.

## 4. Conclusions

Carbon nitride was successfully supported on TiO_2_/hectorite by a high-temperature solid phase reaction and the synthesized g-C_3_N_4_/TiO_2_/hectorite Z-scheme composites with Vo and Ti^3^^+^ have great visible light photo-catalytic activity. The high adsorption ability, great light harvesting and enhanced e^−^-h^+^ separation efficiency are conducive to photoactivity. The formation of Vo and Ti^3+^ and the construction of the Z-type heterojunction promote the photo absorption property and the separation of photogenerated e^−^ and h^+^. Moreover, the composites exhibited good recycle reusability. Owing to excellent photoactivity and good reusability, the g-C_3_N_4_/TiO_2_/hectorite composites have good potential for the photo-degradation of organic pollutants as visible light photo-catalysts. 

## Figures and Tables

**Table 1:** The relative atomic content of surface elements for g-C_3_N_4_/TiO_2_/hectorite reused before and after measured by y X-ray photoelectron spectroscopy (XPS).

Sample	Ti (%)	Si (%)	Mg (%)	N (%)	O (%)	C (%)
g-C_3_N_4_/TiO_2_/hectorite	2.07	3.70	2.09	36.52	16.89	38.74
recycled g-C_3_N_4_/TiO_2_/hectorite ^1^	3.44	6.15	3.99	31.73	27.37	27.31

^1^ It was reused for five times.

**Table 2:** The specific surface area, pore volume, pore diameter and crystallite size of TiO_2_.

Sample	Specific Surface Area ^1^ (m^2^/g)	Pore Volume ^2^ (cm^3^/g)	Average Pore Radius ^3^ (nm)	Crystallite Size of TiO_2_^4^ (nm)
Hectorite	70.0005	0.102052	4.8226	-
TiO_2_/hectorite	228.8244	0.174202	3.2794	11.0
g-C_3_N_4_/TiO_2_/hectorite	219.0311	0.360411	5.8204	11.3
g-C_3_N_4_	103.0738	0.185361	5.9241	-
P25	50	-	-	21

^1^ The specific surface area is calculated by Brunauer-Emmett-Teller (BET) method and the correlation coefficient is up to 0.9999. ^2,3^ The pore volume and pore radius are calculated by Barrett-Joyner-Halenda (BJH) method according to the absorption branch. ^4^ The crystallite size of TiO_2_ is calculated by the Debye-Scherrer equation according to the 101 peak.
